# Supplementary material for: Structural basis for ion selectivity in TMEM175 K+ channels
Source: eLife. 2020 Apr 8;9:e53683. doi: 10.7554/eLife.53683 (PMC7176437; doi:10.7554/eLife.53683)
Supplement: Supplementary file 1 [file elife-53683-supp1.odt]

|  | Native | K+/S | Cs+ | Rb+ | Zn2+ |
| --- | --- | --- | --- | --- | --- |
|  |  |  |  |  |  |
| PDB Code | 6HD8 |  | 6HDA | 6HD9 | 6HDB |
|  |  |  |  |  |  |
| Data collection |  |  |  |  |  |
| Wavelength (Å) | 0.87933 | 2.02460 | 2.00690 | 0.79480 | 1.24610 |
| Space group | P4 21 2 | P4 21 2 | P4 21 2 | P4 21 2 | P4 21 2 |
| Unit cell dimensions |  |  |  |  |  |
| a, b, c (Å) | 131.2 131.2 132.6 | 132.0 132.0 132.6 | 131.7 131.7 131.5 | 130.5 130.5 132.4 | 131.9 131.9 132.7 |
| α, ß, λ (°) | 90, 90, 90 | 90, 90, 90 | 90, 90, 90 | 90, 90, 90 | 90, 90, 90 |
| Resolution (Å) | 46.6-2.4 | 46.8-3.5 | 46.5-3.8 | 46.5-3.5 | 46.64-2.90 |
|  | (2.49-2.4)* | (3.63-3.5)* | (3.94-3.8)* | (3.62-3.5)* | (3.0-2.9)* |
| Rmeas (%) | 29.1(415.3) | 6.8 (128.3) | 24.8 (776.8) | 27.9 (351.2) | 17.7(1,432) |
| Rpim (%) | 2.2 (43.1) | 1.8 (30.3) | 1.9 (41.7) | 3.3 (74.0) | 2.6 (223.0) |
| I/σI | 22.7(1.5) | 15.6 (2.0) | 10.2 (1.0) | 11.4 (1.4) | 10.2 (0.60) |
| CC1/2 | 100.0 (49.4) | 99.6 (88.6) | 100.0 (75.7) | 100.0 (51.1) | 99.9 (46.7) |
| Completeness (%) | 99.3 (99.4) | 99.8 (98.3) | 99.9 (100.0) | 99.3 (93.2) | 99.9 (99.8) |
| Redundancy | 166.6(83.2) | 9.6 (9.3) | 40. (40.0) | 27.7 (23.0) | 27.0 (27.1) |
| Unique reflections | 45,710 (4500) | 28,188 (2080) | 11,875 (1153) | 14,836 (1400) | 26,575 (2,588) |
|  |  |  |  |  |  |
| Refinement |  |  |  |  |  |
| Resolution (Å) | 24.16 – 2.40 |  | 30.21 – 3.80 | 21.47 – 3.50 | 44.1 – 2.90 |
| Rwork/Rfree | 0.209/0.253 |  | 0.280/0.337 | 0.289/0.291 | 0.275/0.286 |
| Ramachandran |  |  |  |  |  |
| favored/outlier | 95.9/0.0 |  | 94.0/0.3 | 95.9/0.0 | 95.3/0.2 |
| No. atoms | 5,670 |  | 5,549 | 5,532 | 5,548 |
| Protein | 5,463 |  | 5,463 | 5,463 | 5,463 |
| Ligand/ion | 55 |  | 54 | 54 | 56 |
| Water | 152 |  | 32 | 15 | 29 |
| B-factors | 132.7 |  | 170.2 | 173.3 | 161.8 |
| Protein | 134.1 |  | 171.0 | 174.2 | 162.2 |
| Ligand/ion | 91.7 |  | 93.5 | 93.7 | 141.5 |
| Water | 95.1 |  | 158.7 | 156.0 | 124.6 |
| R.m.s. deviations |  |  |  |  |  |
| Bond lengths (Å) | 0.014 |  | 0.013 | 0.016 | 0.013 |
| Bond angles (°) | 1.57 |  | 1.49 | 1.82 | 1.52 |
| Clashcore | 0.54 |  | 1.36 | 1.27 | 0.09 |

*Values in parentheses are for highest-resolution shell.

Supplementary file 1. Crystallographic data collection and refinement statistics. The resolution cutoff was determined by CC1/2 criterion. For the native data set seven datasets from a single crystal were merged together. For the K+/S data set, two sets from a single crystal have been merged together. For the Cs+ dataset, three datasets from three crystals have been merged together. Six data sets from two crystals have been merged together for the Rb+ data set. For the Zn2+ data set, two data sets from two crystals have been merged together.
