# Supplementary material for: Structural basis for ion selectivity in TMEM175 K+ channels
Source: eLife. 2020 Apr 8;9:e53683. doi: 10.7554/eLife.53683 (PMC7176437; doi:10.7554/eLife.53683)
Supplement: Supplementary file 2 [file elife-53683-supp2.odt]

|  | T38A_Native | T38A_Zn2+ |  |
| --- | --- | --- | --- |
|  |  |  |  |
| PDB Code | 6HDC | 6SWR |  |
|  |  |  |  |
| Data collection |  |  |  |
| Wavelength (Å) | 1.00004 | 1.280961 |  |
| Space group | P4 21 2 | P21 2 21 |  |
| Unit cell dimensions |  |  |  |
| a, b, c (Å) | 133.5 133.5 132.5 | 129.8  131.6 151.8 |  |
| α, ß, λ (°) | 90, 90, 90 | 90, 90, 90 |  |
| Resolution (Å) | 47.2- 3.40 | 49.7-3.2 |  |
|  | (3.67-3.40)* | (3.32-3.2)* |  |
| Rmeas (%) | 255.2 (10,621) | 48.0 (17,764) |  |
| Rpim (%) | 21.6 (901.5) | 6.8 (254.3) |  |
| I/σI | 6.5 (1.4) | 6.5 (0.5) |  |
| CC1/2 | 99.9 (79.7) | 99.3 (66.6) |  |
| Completeness (%) | 99.9 (99.6) | 99.7 (99.8) |  |
| Redundancy | 146.0 (144.6) | 51.8 (47.2) |  |
| Unique reflections | 16,953 (3435) | 43,391 (4510) |  |
|  |  |  |  |
| Refinement |  |  |  |
| Resolution (Å) | 22.25– 3.40 | 28.62-3.2 |  |
| Rwork/Rfree | 0.273/0.292 | 0.269/0.299 |  |
| Ramachandran |  |  |  |
| favored/outlier | 95.9/0.0 | 95.7/0.37 |  |
| No. atoms | 5,542 | 10,962 |  |
| Protein | 5,461 | 10,806 |  |
| Ligand/ion | 54 | 107 |  |
| Water | 27 | 49 |  |
| B-factors | 178.9 | 223.8 |  |
| Protein | 179.6 | 224.4 |  |
| Ligand/ion | 150.7 | 201.8 |  |
| Water | 101.8 | 138.9 |  |
| R.m.s. deviations |  |  |  |
| Bond lengths (Å) | 0.016 | 0.016 |  |
| Bond angles (°) | 1.81 | 1.72 |  |
| Clashcore | 0.99 | 2.84 |  |

*Values in parentheses are for highest-resolution shell.

Supplementary file 2. Crystallographic data collection and refinement statistics. The resolution cutoff was determined by CC1/2 criterion. For the datasets of the T38A mutant and the T38A mutant soaked with Zn2+, four datasets from two crystals have been merged together each.
