# Supplementary material for: Structural basis for ion selectivity in TMEM175 K+ channels
Source: eLife. 2020 Apr 8;9:e53683. doi: 10.7554/eLife.53683 (PMC7176437; doi:10.7554/eLife.53683)
Supplement: Transparent reporting form [file elife-53683-transrepform.docx]

***eLife’s* transparent reporting form**

We encourage authors to provide detailed information *within their submission* to facilitate the interpretation and replication of experiments. Authors can upload supporting documentation to indicate the use of appropriate reporting guidelines for health-related research (see [EQUATOR Network](http://www.equator-network.org/%20)), life science research (see the [BioSharing Information Resource](https://biosharing.org/)), or the [ARRIVE guidelines](http://www.plosbiology.org/article/info:doi/10.1371/journal.pbio.1000412) for reporting work involving animal research. Where applicable, authors should refer to any relevant reporting standards documents in this form.

If you have any questions, please consult our Journal Policies and/or contact us: [editorial@elifesciences.org](mailto:editorial@elifesciences.org).

**Sample-size estimation**

- You should state whether an appropriate sample size was computed when the study was being designed
- You should state the statistical method of sample size computation and any required assumptions
- If no explicit power analysis was used, you should describe how you decided what sample (replicate) size (number) to use

Please outline where this information can be found within the submission (e.g., sections or figure legends), or explain why this information doesn’t apply to your submission:

-Electrophysiological measurements were repeated to the extent that additional

replicates did not substantially alter the average values. The number of biological

replicates ranged from 3 to 24 as indicated in figure legends. Errors are

shown as SD for the indicated number of replicates as indicated in figure

legends.

-Numbers of replicates are according to common standards in the research field.

**Replicates**

- You should report how often each experiment was performed
- You should include a definition of biological versus technical replication
- The data obtained should be provided and sufficient information should be provided to indicate the number of independent biological and/or technical replicates
- If you encountered any outliers, you should describe how these were handled
- Criteria for exclusion/inclusion of data should be clearly stated
- High-throughput sequence data should be uploaded before submission, with a private link for reviewers provided (these are available from both GEO and ArrayExpress)

Please outline where this information can be found within the submission (e.g., sections or figure legends), or explain why this information doesn’t apply to your submission:

-Number of replicates is indicated in each respective figure legend panel. A

minimum of three replicates was performed, in most figure data more than 4-10

and up to 24 datapoints were acquired for histograms.

- All replicates in electrophysiology experiments are biological, which means that

single datapoints were acquired from individual transfected cells.

-All transfections for electrophysiological recordings were repeated with similar

results.

-No obvious outliers related to electrophysiological experiments were omitted

from figure data. Only outliers resulting from technical complications, such as

leaky membrane seals in patch clamp experiments or transfections with low

efficiency, were discarded.

-Detailed statistics for crystallography are indicated in the Methods section under

“Data collection and Structure Determination” and in the provided crystallographic

tables (Supplementary tables 1 and 2).

-All presented data is according to eLife’s regulations and information regarding the

number of replicates is provided in figure legends, materials section or the main text.

-Biochemical data from Arg24-mutant proteins (size exclusion chromatography in

supplementary Fig 1h) is from a single experiment.

**Statistical reporting**

- Statistical analysis methods should be described and justified
- Raw data should be presented in figures whenever informative to do so (typically when N per group is less than 10)
- For each experiment, you should identify the statistical tests used, exact values of N, definitions of center, methods of multiple test correction, and dispersion and precision measures (e.g., mean, median, SD, SEM, confidence intervals; and, for the major substantive results, a measure of effect size (e.g., Pearson's r, Cohen's d)
- Report exact p-values wherever possible alongside the summary statistics and 95% confidence intervals. These should be reported for all key questions and not only when the p-value is less than 0.05.

Please outline where this information can be found within the submission (e.g., sections or figure legends), or explain why this information doesn’t apply to your submission:

-All electrophysiology data is presented with the exact number of replicates and

dispersion of the values around the mean value is given as SD.

(For large datasets, or papers with a very large number of statistical tests, you may upload a single table file with tests, Ns, etc., with reference to sections in the manuscript.)

**Group allocation**

- Indicate how samples were allocated into experimental groups (in the case of clinical studies, please specify allocation to treatment method); if randomization was used, please also state if restricted randomization was applied
- Indicate if masking was used during group allocation, data collection and/or data analysis

Please outline where this information can be found within the submission (e.g., sections or figure legends), or explain why this information doesn’t apply to your submission:

N/A

**Additional data files (“source data”)**

- We encourage you to upload relevant additional data files, such as numerical data that are represented as a graph in a figure, or as a summary table
- Where provided, these should be in the most useful format, and they can be uploaded as “Source data” files linked to a main figure or table
- Include model definition files including the full list of parameters used
- Include code used for data analysis (e.g., R, MatLab)
- Avoid stating that data files are “available upon request”

Please indicate the figures or tables for which source data files have been provided:

Additional source data files are provided for the following figures.

Electrophysiological measurements:

Fig. 2; Figure 2-figure supplement 1a; Fig. 5 b,d; Fig. 6; Figure 6-figure supplement 1a

Crystallographic analysis:

Figure 1-figure supplement 4

Biochemical experiments:

Figure 2-figure supplement 4b
